# INTEGRA study protocol: primary care intervention in type 2 diabetes patients with poor glycaemic control

**DOI:** 10.1186/s12875-019-0916-9

**Published:** 2019-02-07

**Authors:** Àngels Molló, Anna Berenguera, Esther Rubinat, Bogdan Vlacho, Manel Mata, Josep Franch, Bonaventura Bolíbar, Dídac Mauricio

**Affiliations:** 1 Centre d’Atenció Primària de Cervera, Lleida, Spain; 2 Insitut Català de la Salut. Institut Universitari d’Investigació en Atenció Primària Jordi Gol, Grup DAP_CAT.), Barcelona, Spain; 3 Membre del Grup d’Estudi de la Diabetis en Atenció Primària (GEDAPS) de la Societat Catalana de Medicina Familiar i Comunitària (CAMFIC) i de la RedGDPS, Barcelona, Spain; 4 Institut Universitari d’Investigació en Atenció Primària Jordi Gol, Barcelona, Spain; 5 Universitat Autònoma de Barcelona, Bellaterra, Cerdanyola del Vallès, Spain; 6 Institut Universitari d’Investigació en Atenció Primària Jordi Gol, Barcelona, Spain; 7 Serra Húnter fellow - Facultad de Enfermería y Fisioterapia de la Universidad de Lleida, Lleida, Spain; 8 Grup de Recerca en Cures de la Salut (GRECS) – IRBLleida, Lleida, Spain; 9 Centro de Investigacion Biomedica en Red de Diabetes y Enfermedades Metabolicas Asociadas (CIBERDEM), Instituto de Salud Carlos III (ISCIII), Gran Via Corts Catalanes, 587, àtic, 08007 Barcelona, Spain; 10 Institut Universitari d’Investigació en Atenció Primària Jordi Gol, Gran Via Corts Catalanes, 587, àtic, 08007 Barcelona, Spain; 11 Centre d’Atenció Primària La Mina, Barcelona, Spain; 12 CIBER of Diabetes and Associated Metabolic Diseases (CIBERDEM), Madrid, Spain; 13 Institut Català de la Salut, Cerdanyola del Vallès, Spain; 14 Institut Universitari d’Investigació en Atenció Primària Jordi Gol, Barcelona, Spain; 15 Membre del Grup d’Estudi de la diabetis en Atenció Primària (GEDAPS) de la Societat Catalana de Medicina Familiar i Comunitària (CAMFIC) i de la RedGDPS, C. Mar, s/n 08930 Sant Adrià de Besòs, Barcelona, Spain; 16 Centre d’Atenció Primària Raval Sud, CIBER of Diabetes and Associated Metabolic Diseases (CIBERDEM), Institut Català de la Salut, Institut Universitari d’Investigacióen Atenció Primària Jordi Gol, Av. Drassanes, 17-21 08001 Barcelona, Spain; 17 Institut Universitari d’Investigacióen Atenció Primària Jordi Gol, Barcelona, Spain; 18 Universitat Autònoma de Barcelona, Campus de la UAB, Plaça Cívica, 08193 Bellaterra, Spain; 19 Department of Endocrinology & Nutrition, CIBER of Diabetes and Associated Metabolic Diseases (CIBERDEM), Health Sciences Research Institute and University Hospital de la Santa Creu i Sant Pau, Barcelona, Spain; 20 Institut Universitari d’Investigació en Atenció Primària Jordi Gol, Gran Via de Les Corts Catalanes, 591 atico, 08007 Barcelona, Spain; 21 Department of Endocrinology & Nutrition, Hospital de la Santa Creu i Sant Pau, Sant Quinti, 89, 08041 Barcelona, Spain

**Keywords:** Glycated haemoglobin, Clinical inertia, Intervention, Primary care, Type 2 diabetes, Treatment intensification

## Abstract

**Background:**

The management of hyperglycaemia and associated cardiovascular risk factors in patients with type 2 diabetes mellitus (T2DM) may reduce diabetes-related complications. The strategy to broaden the knowledge base of primary care professionals to improve health care has mainly been prompted by the current reality of limited resources and access to specialized care. The main objective of this study is to assess the effectiveness of comprehensive interventions focused on treatment intensification, decrease clinical inertia and reduce possible barriers to treatment adherence in patients with poorly controlled diabetes in a primary care setting.

**Methods:**

This is a two-phase mixed method study, whose aims are the development of complex interventions and the assessment of their effectiveness. The main study outcome is a change in glycated haemoglobin (HbA1c) levels.

The INTEGRA study is divided into two phases. Phase 1: A qualitative study with a phenomenological approach using semi-structured interviews with the objective of determining the factors related to the participants and health care professionals that influence the development and implementation of a specific intervention strategy aimed at patients with poor glycaemic control of T2DM in primary care. Phase 2: Exploratory intervention study to be conducted in Primary Health Care Centres in Catalonia (Spain), including 3 specific health care areas.

The intervention study has two arms: Intervention Group 1 and 2. Each intervention group will recruit 216 participants (the same as in the control group) between the ages of 30 and 80 years with deficient glycaemic control (HbA1c > 9%). The control group will be established based on a randomized selection from the large SIDIAP (Sistema d’Informació per al desenvolupament de la Investigació en Atenció Primària) database of patients with comparable socio-demographic and clinical characteristics from the three provinces.

**Discussion:**

This study is a comprehensive, pragmatic intervention based on glycaemic treatment intensification and the control of other cardiovascular risk factors. It is also aimed at improving treatment adherence and reducing clinical inertia, which could lead to improved glycaemic control and could likewise be feasible for implementation in the actual clinical practice of primary care.

**Trial registration:**

Clinicaltrials.gov. registration number. NCT02663245; January 25, 2016.

**Electronic supplementary material:**

The online version of this article (10.1186/s12875-019-0916-9) contains supplementary material, which is available to authorized users.

## Background

Type 2 diabetes mellitus (T2DM) is a chronic disease with a significant socioeconomic impact due to its high prevalence, the impact of associated complications, and high rate of mortality [1]. The elevated costs associated with the disease and its impact on patient quality of life are currently the target of many health plans and government strategies. The benefits of proper control of T2DM and the related cardiovascular risk factors, especially blood pressure and lipids, are widely accepted [2]. However, studies in Spain show that although evidence of the improved care of T2DM patients exists, the therapeutic goals are often not achieved in real-life clinical practice [3, 4].

A meta-analysis performed by Tricco et al. regarding the evaluation of strategies used in clinical trials for the improvement of quality procedures used in the management of diabetes shows evidence that the most notable improvements are observed when baseline levels of glycated haemoglobin (HbA1c) are high, especially over 8%, in diabetic patients [5]. This suggests that interventions should focus on patients with poorer control. With regard to strategies aimed at professionals and organizations that demonstrated efficacy, the most efficient were feedback from the information obtained in audits, training of professionals, and organizational changes (such as electronic records, clinical reminders, and case management, in addition to financial incentives).

Treatment adherence and clinical inertia play an important role in cases of insufficient glycaemic control. Therapeutic or clinical inertia is defined as the failure to initiate or to intensify treatment when indicated [6], thereby preventing or delaying the benefits of proper control. In the DIAMOND [7] study conducted in Spanish primary care centres, mean HbA1c was 8.1% over an average of 2.9 years, with values of > 7% before change, when switching from monotherapy to combination therapy was observed. Additionally, in an assessment of the GEDAPS group carried out in Catalonia, clinical inertia was detected in 33% of patients, and the mean HbA1c level to perform the treatment change was 8.4%. [8].

Sometimes, clinical inertia is related to the non-compliance of the patient [3, 9]. As seen in the Grant et al. [10] study, interventions oriented to improve patient adherence could consequently decrease clinical inertia. However, there are few studies on interventions aimed specifically at reducing clinical inertia.

Feedback to professionals is another possible key factor for improving clinical inertia. In an American study [11, 12], feedback was given to the professionals on the objectives achieved via alerts in the electronic records and/or weekly short meetings between professionals. As a result, treatment intensification increased from 35 to 52% in 3 years, and a significant improvement in HbA1c values (in the group receiving feedback, mean HbA1c was 7.5% vs. 8.2% in the control group) was also observed. However, in the Rochester study [13], although a reduction of more than 50% was observed in clinical inertia, this did not translate into an improvement in the control parameters after one year. Therefore, although interventions can be effective in reducing clinical inertia, they are not always effective in improving metabolic control.

Thus, there is a need to design strategies that help achieve the desired treatment goals that can be simultaneously implemented in real-life clinical practice in the primary care setting and the best way to achieve this goal is to use the mixed methodology by previously exploring the patients’ own perspective, in order to design a proper implementation strategy. Previous studies have shown that specialised Diabetes Unit improve glycaemic control [11]. Our main intervention was designed to evaluate if a local monographic consultation run by primary healthcare professionals could be effective in the context of real-world primary healthcare practice for the management of very poor controlled diabetic patients.

## Methods/design

### Aims of the study

Following this line of thought, the aim of this study is to assess whether glycaemic control improves in very poorly controlled type 2 diabetic patients as a result of interventions based on treatment intensification and on an increase in the patients’ adherence to treatment with a diabetes-targeted clinic specifically held for those patients.

The hypothesis is that in patients with inadequately controlled T2DM, a preliminary comprehensive detection intervention developed based on a monographic consultation interview and a complex intervention to improve the self-efficacy of patients could improve the effectiveness and cost-effectiveness in controlling glycaemia and other metabolic and risk factor parameters compared to the usual practice.

The secondary aims are to determine:

- The percentage of patients who achieve HbA1c levels of < 7% and < 8%.
- The improvement of lipidaemic control measured by the mean concentration of low-density lipoprotein cholesterol(LDL-C), non-high density lipoprotein cholesterol (non-HDL-C), and triglycerides in the intervention groups relative to the control group.
- Whether the percentage of patients who achieve target levels of LDL-C, non-HDL-C, and triglycerides recommended by the Catalan Health Institute guidelines (REGICOR tables) [14] is higher in the intervention groups than in the control group.
- Whether the percentage of diabetic patients with mean systolic blood pressure < 140 mmHg and mean diastolic blood pressure < 90 mmHg is higher in the intervention groups than in the control group.
- Whether the adherence to the screening for chronic complications associated with T2DM, according to the protocol of the Catalan Health Institute (fundoscopy, microalbuminuria, examination of the foot: arterial index and peripheral sensitivity), is better in patients who have participated in the specialized consultation unit than in the control group.
- Whether the patient’s self-efficacy to implement changes in risk factors is different in intervention group 1 (IG-1) than in intervention group 2 (IG-2).
- Whether the direct health costs of T2DM patients who participated in the monographic consultation are different from those in the control group.
- The impact on satisfaction and quality of life of patients.
- Whether the improvement in the control of behavioural cardiovascular risk factors (smoking and exercise) is better in the intervention than in the control groups.
- The improvement in the adequacy and intensification of treatment (clinical inertia).

### Study design

A mixed method study will be carried out, which will compare poorly controlled T2DM patients (poor glycaemic control was defined as an HbA1c value > 9% in the last test performed in the 12 months prior to study inclusion), treated with two different comprehensive approaches with a control group receiving only usual clinical care. The mixed methodology for the development of complex interventions composed of two phases: a first phase consisting of a qualitative study carried out through personal interviews with patients and professionals, and a second quantitative phase that will correspond to the execution of the clinical trial and in which the findings of the first phase will be applied.

We designed this study considering that the intervention group 2 will be the control group for Intervention group 1, where the specific monographic consultation is implemented. Group Intervention 1 includes the main intervention for which we will test the effectiveness to improve glycaemic control. In parallel to Intervention group 2, we decided to include an additional comparison group consisting of type 2 diabetic subjects attending primary care centres managed by our institution in our region, with subjects selected according to the same study criteria. To select this latter group, we describe the use of the Sistema d´Informació per al Desenvolupament de la investigació en Atenció Primària (SIDIAP) database, which contains anonymised electronic health records of patients attended at primary care centers of the same health care districts not participating in the study.

This study is non-randomized because it would be difficult give a different treatment to an individual in the same primary care center treated by the same professional without it affecting the outcome in the standard care arm of the study.

### Study setting

This mixed method study will be conducted in primary care settings in 3 different health care territories of the Catalan Health Institute (Lleida, Barcelona, and Girona). Each basic health care area will be selected with similar characteristics and will be organized into the intervention group 1 (IG-1), the intervention group 2 (IG-2), and a control group.

### Characteristics of participants

The inclusion criteria include the following: T2DM of more than one year of disease duration, age between 30 and 80 years, HbA1c > 9% in the last test performed in the 12 months prior to study inclusion, with no change in antidiabetic treatment within the previous 3 months. The changes in the dose of a drug in the antidiabetic treatment will be not considered as exclusion criteria.

The exclusion criteria include the following: T2DM controlled by endocrinologist at the moment of inclusion, systemic glucocorticoid treatment (ATC code: H02AB) or orlistat treatment (ATC code: A08AB01) (chronic or during the two months prior to inclusion), estimated life expectancy < 2 years, active treatment for malignancy (other than basal cell cancer or squamous cell skin cancer), serious mental disorders (psychosis, bipolar affective disorder, severe depressive episode with psychotic symptoms), class III or IV (New York Heart Association functional classification) heart failure (ICD-10: I50), dementia, renal transplantation or current dialysis treatment, history of drug and/or alcohol abuse (ICD-10: F10-F14), pregnancy or lactation, treatment with immunosuppressive drugs, haemoglobinopathies (ICD-10: D58.2), or chronic anaemia, body mass index (BMI) > 45 kg/m^2^, or any conditions that the investigator considered could prevent the patient from completing the study.

### Study phases

The INTEGRA study is divided into two distinct phases. Phase 1 is a qualitative research study, which is aimed at identifying viable strategies to overcome barriers to treatment; these strategies have been included in the intervention study (phase 2). Phase 2 is a controlled, non-randomized, interventional, pragmatic study that involves primary care centres in 3 regions in Catalonia (Lleida, Girona and Barcelona).

#### Phase 1: Qualitative study

##### Design

A qualitative study with a phenomenological approach was conducted to identify the barriers and facilitators for the management of poorly controlled T2DM patients. This approach was taken in order to identify psychosocial factors influencing glycaemic control. Briefly, we collected information on the patient’s perception on the communication of the diagnosis, cognitive representation of the disease (knowledge, cause, symptoms, duration, consequences and control of the disease), emotions associated with the disease (e.g., fears or worries about the future), and their cognitive and emotional attitudes regarding strategies to control diabetes (diet, physical activity, and pharmacological activity). Finally, patients described their perceived relationship with the health-care professionals and gave their input regarding the design of the INTEGRA study (e.g., visit schedule or use of information and communication technologies). The results of this phase of the study were used to draw recommendations and to design strategies in order to optimize the patient’s adherence and disease control during the intervention phase of the INTEGRA study. Thus, other components that may be needed for the intervention, such as the use of Information and Communication Technologies like internet platforms or mobile applications, was also determined.

##### Recruitment strategies and participants

At each primary care centre, a liaison was selected to facilitate the communication between the centre and the research team. One member of the research team contacted the liaison at each primary care centre. These liaisons, together with the primary care professionals, identified the patients who meet the criteria of poor glycaemic control and contacted the potential participants.

##### Sampling

The sampling for the study was opportunistic [15]. Although not a theoretical sample, it was taken into account the following variables: gender, age, years of progression of T2DM, and type of treatment (oral antidiabetics, insulin, or a combination).

##### Techniques for generating information

A psychologist carried out 8 to 10 semi-structured individual interviews in each Primary Health Care Centre. This interview technique is especially useful when it is important to collect the subjective opinions of societal actors, and becomes even more valuable when wanting to explore diverse points of view that represent the different attitudes that might exist regarding the subject of investigation [16]. The interviews were audio-recorded and subsequently transcribed systematically and verbatim.

##### Analysis

A thematic content analysis was performed based on the information obtained from the interviews [17]. The data was analysed in the following manner: after successive readings of the transcribed interviews [18], the researchers attained some pre-analytical insight into the data. Next, four researchers conducted the following analytical steps: (a) identification of the relevant subjects and texts; (b) fragmentation of the text into units of meaning; (c) text codification with a mixed strategy: Leventhal model and emerging codes from the data; (d) creation of categories by grouping the codes based on the criterion of similarity; (e) analysis of each category; and (f) elaboration of a new text with the results. These results were subsequently discussed by the research team members until a consensus was reached (triangulation).

The results of the qualitative study have been published recently [19].

#### Phase 2: Interventional study

##### Design

A controlled, non-randomized interventional study that involves primary care centres of 3 health care districts in Catalonia (Lleida, Girona, and Barcelona).

##### Participants

The participants are T2DM patients with poor glycaemic control that meet all the inclusion criteria and sign the informed consent.

##### Inclusion criteria for primary health care Centres

The participating Primary Care centres must meet the following criteria to be eligible: I) experience of more than 5 years using the eCAP™ programme (electronic clinical records of Primary Care Centres in Catalonia) to optimize the use of the software as a source of information and intervention (warnings, alerts, etc.); II) centres with more than 10 general practitioners, to obtain a large number of study subjects; III) centres belonging to different health care areas, to increase external validity of the study; and IV) acceptance by the majority of professionals in the centre.

##### Intervention design

In each basic health area, two intervention groups and one control group were included. Two different interventions focused on treatment intensification, namely reducing inertia by professionals and reducing the possible barriers to treatment adherence have been implemented, one in each intervention group. In order to reduce inertia by primary care professionals, to get closer to the objectives of health control, and to allow reproducibility in the current context of primary care centers, the intervention was carried out through multiple integrated strategies. To improve the intervention replicability, the Template for Intervention Description and Replication guide was followed [20]. Each health care district has a contact in a hospital with a Department of Endocrinology who provides specialized support for professionals if needed.

#### Intervention group 1 (IG-1)

Intervention 1 consists of a comprehensive strategy with the following components:

- A specialized consultation carried out by a general practitioner and a nurse that will provide the participant with a complete assessment of their case, and a series of recommendations will be set for case management, together (only if required) with the specialized team from Endocrinology and Nutrition using a telematics communication system. The strategy recommendation includes management measures aimed at optimizing blood glucose, blood pressure, lipid profile, and other cardiovascular risk factors, as well as the detection of diabetes-associated complications following the guidelines of the Catalan Health Institute [21] and the guidance document of the Department of Health of the local government (Generalitat de Catalunya) [22].
- Basic training in clinical practice guidelines: consulting professionals will participate in workshops related to clinical practice guidelines, thus broadening the professionals’ knowledge base with regard to diabetes by providing them with an increased degree of autonomy in diabetes case management.
- Training of professionals in coaching: all professionals in the primary care centres will participate in a 7-h training programme for coaching to be able to impart practical theoretical content on the following subjects: strategies for active listening; strategies for communication without value judgement; support strategies to develop self-management skills for diabetes, hypertension, and hyperlipidaemia; strategies to provide social and emotional support; strategies to motivate lifestyle changes; strategies for medication adherence; and strategies to access community resources.
- The professionals will attend a 2-h training programme to update their training for reviewing the practical cases discussed in consultation using a coaching strategy point of view.
- Interventions based on patients’ SMS phone messages (sending a message 1 time / week during the first three months) to promote the change in behaviour and to maintain healthy lifestyle changes to improve diabetes control.

#### Intervention group 2 (IG-2)

**Fig. 1:**
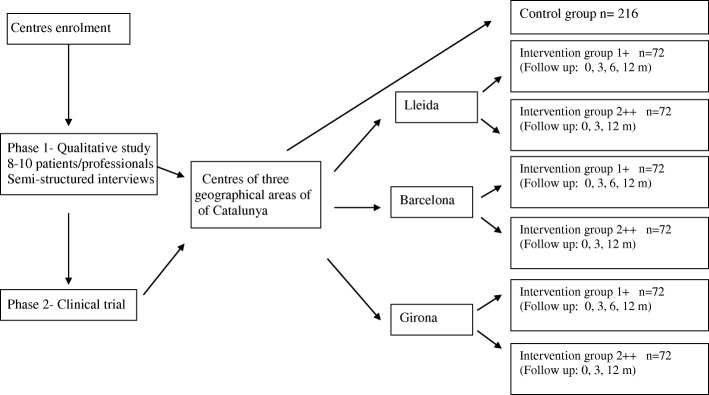
Study flow chart. *Control group: usual clinical care with the usual control by the family doctor and nurse according to the current CPG protocol. +Intervention 1: Usual clinical care with the usual control + Monographic consultation + Basic training in clinical practice guidelines + Training in coaching + 2-h training program to update the coaching strategy + Intervention based on patients SMS phone messages. ++Intervention 2: Usual clinical care with the usual control + Basic training in clinical practice guidelines + Training in coaching + 2-h training program to update the coaching strategy + Intervention based on patients SMS phone messages. m: months

Patients allocated to the IG-2 will receive the same personalized intervention described for the IG-1, without the monographic consultation (Fig. 1).

#### Control group

The control group will consist of a randomized selection of patients who meet the inclusion criteria and have the same sociodemographic and clinical characteristics as the patients from the intervention groups, including the three participating health care districts. Patients allocated in this group will receive routine clinical care with the usual control by the family doctor and nurse according to the current Catalan Health Institute protocol. The data of patients in the control group will be obtained from the electronic medical record (SIDIAP) database [23]. The inclusion period for the control group will be from 9 December 2015 (the date when the first patient entered the study) until 31 October 2017 (end of the recruiting period). Data on diabetic status, glycaemia and all other study variables that were recorded in the electronic health records for patients in the intervention groups will be extracted from the SIDIAP database for 12 months for each patient. Participants will be followed up until they experience the outcomes of interest, die, leave the SIDIAP database (e.g., change of address) or complete the follow-up (31 October 2018). If the intervention groups reach the study sample earlier than 31 July 2017, the data for the control group will be followed until 12 months from the date when the last patient would be enrolled in the intervention group.

##### Study variables

The variables are collected at different time-points during the study (Table 1). These include: sociodemographic variables (age, sex), anthropometric variables (height, weight, BMI, blood pressure), clinical variables (drugs and dosages), smoking habits (smoking, non-smoker, ex-smoker), presence of multiple disorders (Clinical Risk Group and identified in the computerized patient clinical history and the number of health problems as a proxy asset of multi-morbidity), presence of microvascular complications (diabetic retinopathy, diabetic nephropathy, and diabetic neuropathy), presence of macrovascular complications (ischaemic heart disease, stroke, peripheral vascular disease, and heart failure), laboratory variables according to the standard procedures of the laboratory of reference (HbA1c, blood count, renal function- creatinine, glomerular filtration rate, urinary excretion of albumin), liver function (Alanine Aminotrasferase (ALT), Aspartate Aminotransferase (AST), alkaline phosphatase, Gammaglutamil transferase (GGT)), lipid profile (total cholesterol, HDL-C, triglycerides, LDL-C, and non-HDL-C), and episodes of severe hypoglycaemia (episodes of hypoglycaemia that require the intervention of a third party for recovery will be recorded).

**Table 1:** Study timeline, variables and procedures

	IG-1 and IG-2					Control group	
Data collected	Assessment (months)				Source	Assessment	Source
	0	3	6	12		Month 0 & Month 12	
Inclusion/Exclusion criteria	1 + 2	1 + 2	1	1 + 2	e-CRF	§	SIDIAP
Sociodemographic variables and comorbidities: *1. Age, height, gender, weight, BMI, duration of T2DM, medication (active component and dose),* etc.*...* *2. Pluripathology, clinical risk group*	1 + 2	1	1	1 + 2	eCAP	§	SIDIAP
Main variable: HbA1c	1 + 2	1	1	1 + 2	e-CRF eCAP	§	SIDIAP
Lipid profile and other laboratory variables: Total cholesterol, HDL, LDL, triglycerides, non-HDL, urea, creatinine, glomerular filtration rate, urine albumin, AST, ALT, FA, GGT. Systolic and diastolic blood pressure	1 + 2	1	1	1 + 2	eCAP e-CRF	§	SIDIAP
Smoking habit	1 + 2	1	1	1 + 2	eCAP e-CRF	§	SIDIAP
Sedentary lifestyle	1 + 2	1 + 2	1	1 + 2	e-CRF		
Detection of macrovascular complications *Ischaemic heart disease, stroke, peripheral artery disease, heart failure*	1 + 2	1	1	1 + 2	eCAP e-CRF	§	SIDIAP
Chronic microvascular complications detection *Diabetic retinopathy, diabetic nephropathy (albuminuria, renal failure), diabetic neuropathy*.	1 + 2	1	1	1 + 2	eCAP e-CRF	§	SIDIAP
Costs: *Cost of medication, laboratory tests, additional tests, cost of staff (visits), referrals, IT*	1 + 2			1 + 2	eCAP e-CRF	§	SIDIAP
*Costs:* *Urgent care, outpatient and hospital admissions*	1 + 2			1 + 2	e-CRF		
Satisfaction and quality of life of patients *DTSQ / ESDQoL*	1 + 2		1	1 + 2	e-CRF		
Professional Satisfaction *Specific Questionnaire*				1 + 2	e-CRF		
intensification/treatment adherence		1 + 2	1	1 + 2	e-CRF		
Occurrence of severe hypoglycaemia episodes	1 + 2	1	1	1 + 2	e-CRF		SIDIAP
“Patient Activation Measure” questionnaire (instrument for disease self-evaluation)	1 + 2	1 + 2		1 + 2	e-CRF		

*For the IG-1 (monographic + training of professionals + additional measures originated in phase 1), clinic visits will be carried out at baseline, 3, 6, and 12 months. For the IG-2 (training of professionals + additional measures originated in phase 1), clinic visits will be carried out at the time of inclusion (month 0), 3, and 12 months*

Variables related to healthcare costs are also collected and include the costs derived from the medication used (cost per patient per month of medication prescribed according to prices set by the Health Ministry), laboratory tests used (based on prices listed in the Catalan Health Institute service portfolio), supplementary tests, professional costs (the cost of patient visits), cost derived from the referrals to specialists, use of the emergency department, outpatient and hospital admissions, temporary disability, and consumption of testing strips for measuring blood glucose.

The INTEGRA study includes variables evaluated using specific questionnaires:

- Physical activity: this is assessed using the Spanish version of the Brief Physical Activity Assessment Tool questionnaire [24]. It contains two questions that are scored on a scale from 0 to 4. The sum of the scores of both questions are classified as follows: ≥ 4 total score = “sufficiently” active (encourage the patient to continue their activity) or 0 to 3 total score = “insufficiently” active (encourage the patient to increase their activity).
- Patient treatment satisfaction: this is assessed using the Diabetes Treatment Satisfaction Questionnaire (DTSQ) [25] in both the “status” and “change” versions validated for Spanish patients. It comprises 8 sections, each of which is rated on a scale from 0 to 6 points. The score of satisfaction with treatment is the sum of the 6 sections of the questionnaire, which can range from very satisfied (36 points) to very dissatisfied (0 points). The remaining 2 sections measure the perceived frequency of hyperglycaemia and hypoglycaemia from 0 (never perceived) to 6 (perceived most of the time), and they are scored separately.
- Quality of life: this is measured using the validated Spanish version of the Diabetes Quality of Life Questionnaire (ESDQoL) [26], which consists of 21 items, of which 19 relate to specific life domains. The items are scored on a 5-point scale of impact. The effects of diabetes on each of the domains are weighted according to their importance to the quality of life of patients, and the weighted average impact is obtained.
- Satisfaction of the health professionals before and after the interventions proposed in the study is assessed by a questionnaire divided into 4 sections; the first three assess aspects of organization, content, and structure, and the fourth refer to the overall assessment.
- Clinical inertia is evaluated through the questionnaire created by the RedGEDAPS [27]. This questionnaire consists of 5 questions with two possible answers (yes or no) that professionals should answer in each visit when HbA1c > 9%.
- Self-management of the disease is evaluated by the Spanish validated version of the Patient Activation Measure questionnaire [28]. This questionnaire evaluates patients’ ability to play an active role in their health care by assessing knowledge, skills, confidence, and behaviours. It consists of 13 questions in which there are 5 possible response options (strongly disagree, disagree, agree, strongly agree, N/P). The scores range in value from 38.6 to 53.0 (on a 0–100 theoretical point scale).

### Data collection

In the intervention groups (groups 1 and 2), sociodemographic, anthropometric, clinical, and laboratory variables are obtained from the clinical history and anamnesis of the subjects. The data obtained through the questionnaires are collected during the clinic visit according to the subjects’ responses. The variables related to health care costs are obtained from the SIDIAP database. In the control groups, all data will be obtained from the SIDIAP database.

For both groups, variables related to data are collected as described in Table 1 in the Electronic Case Report Form (e-CRF), specifically designed for the purpose of the study.

The consultation with the specialists is carried out in addition to any others that are deemed necessary, and the number and frequency of visits is derived from the interventions proposed to the patients (pharmacological and non-pharmacological).

### Evaluation of the effectiveness between the two intervention groups

The e-CRF has been used to perform an evaluation at baseline, 3, 6, and 12 months. Secondary aims, such as patient’s self-efficacy to implement changes in risk factors, the direct health costs of T2DM patients, and the impact on satisfaction and quality of life of patients, is assessed using the e-CRF data, and the other information will be obtained from SIDIAP database.

### Evaluation of the effectiveness in the three study groups

Data at baseline and at 12 months of the population of all three types of learning centres that meet the defined criteria for inclusion and exclusion will be compared. Criteria will be adequately defined using the ICD-10 codes, thoroughly specifying the temporary windows of the accepted values. Only the outcomes that include SIDIAP variables (as indicated in the Table 1) will be able to be evaluated; even those that are in the e-CRD will not be able to be assessed. This evaluation of effectiveness will be performed in two possible ways: i) following the patients who meet the inclusion criteria in 2015 until the end of the study in 2018 (cohort study), or ii) a cross-sectional study in 2015 and another in 2018.

### Sample size calculation

The sample size was calculated using the GRANMO v.7.12 (IMIM, Barcelona) programme. Considering an alpha risk of 0.05 and a beta risk of less than 0.2 in a bilateral contrast, it was necessary to include 72 patients in each group (intervention and control) to detect a difference equal to or greater than 1% of HbA1c [5] between the two groups. A common standard deviation of 1.46 [6] and a loss rate to follow up of 20% were assumed.

Considering that each physician/nurse that makes up the Basic Care Unit will coordinate their diabetic patients, a correction may be made due to a possible contamination in the analysis between individuals by assigning each Primary Care Team to intervention or control groups and taking into account the design effect. According to a study performed by our research group [16], we estimated that each GP would have 15 potential candidate patients with HbA1c > 9%. Assuming that the intra-class correlation coefficient for Primary Care is 0.05 [29], resulting in a 1.7 design effect, the required sample size will be 42 * 1.7 = 72 individuals in each group.

Each control or intervention group consists of 3 basic health areas, one in each geographical area, each with a mean of 10 BCUs and 15 individuals per basic care unit. Thus, the sample finally calculated to obtain statistical significance was 72 subjects per centre, leading to a total of 648 study subjects.

First patient enrolled in the study INTEGRA on 09/12/2015.

### Statistical analysis plan

For data analysis, the IG-1, the IG-2, and the control group will be compared. All analyses will be carried out using bilateral tests with a nominal significance level of 0.05. However, if the comparisons obtain a *p*-value of less than 0.10, they will also be discussed as indicative of trends. Initially, basal characteristics of all groups will be evaluated to establish homogeneity in age, socio-demographic, comorbidities, laboratory parameters, concomitant medication and diabetes complications. An initial, descriptive comparison between groups of all variables will be performed to evaluate their baseline balance. Statistical significance will be assessed by the chi-square or t-test between the groups. Exploratory analyses will be applied when necessary.

The main variable is set as the difference in mean HbA1c values between the final visit and baseline. The difference will be evaluated in terms of the evolution of HbA1c as contrasted between groups by paired t-Student, or if the assumption of normality is not met for the right non-parametric.

If differences in the characteristics of the patients in each group are observed in the descriptive section of the study, a linear regression of the main variable (parameter evolution during the study) will be adjusted. This linear regression adjusted for the variables identified in the descriptive section, or the ability to adjust the model by setting a propensity score, will be evaluated. For all models, the distribution of waste will be studied, as well as the coefficient of determination (R2), to check for the correct setting.

As was done in Vinagre et al. [4], a subgroup analysis will be performed by a) gender (male / female), b) age: < age 65 / ≥ 65, and c) according to HbA1c control levels (< 10% / ≥ 10%).

We will determine the prevalence of patients who achieved a concentration of HbA1c of less than 7% at the end of the study. A categorical variable (No, Yes) will be calculated. The analysis of a significant decrease in HbA1c will be carried out using a logistic regression analysis adjusting by the effect of the study groups (as *dummy* variable) and the main factors previously determined to be associated with the target of 7% (in a previous descriptive analysis or adjustment by the *propensity score*). The power of discrimination (C statistic and ROC curve) and calibration (Hosmer & Lemeshow test) for the final models will be studied. The software we will use for analysis of the data will be R Core Team (2014). R: A language and environment for statistical computing. R Foundation for Statistical Computing, Vienna, Austria (http://www.R-project.org/).

### Data Management

The researchers who collaborate in each of the primary care centres are responsible for the accurate, complete, and reliable collection of all data. An e-CRF has been created for the study, and a data extraction from patient’s medical history will also be performed. An expert, independent professional from the Primary Care Centres is responsible for periodically monitoring to corroborate the reliability of the data entered in the e-CRF with respect to electronic health records. The detailed description of the study-monitoring plan is shown in the Additional file 1.

### Ethics and dissemination

The study is conducted according to the Helsinki Declaration and Good Clinical Practice guidelines. The study protocol was approved by the Ethics Committee of IDIAP Jordi Gol, Institute of Research in Primary Health Care. Trial Registration Number: P14/129.

Reporting of this trial will adhere to the most relevant and up-to-date CONSORT statement [30] and its relevant extensions [31]. The results from this study will be published regardless of the outcomes.

Confidentiality and anonymity of the data is ensured according to Law 15/1999 of data confidentiality, both in the implementation phase of the project and in the resulting presentations and/or publications. Individual data will be codified to ensure anonymity. Only researchers and monitors will have access to the data.

## Discussion

According to the SIDIAP database, which contains all the data entered into the eCAP, the database used in Catalan Health Institute, 79.6% of the patients have HbA1c values ≤8%, and the proportion of patients with fair control (HbA1c ≤7%) is 56.1% [4].

The INTEGRA study defines potential pragmatic new strategies to improve metabolic control in patients with very poorly controlled T2DM in the primary care setting that, if demonstrated to be effective, could be implemented by professionals in their real-life practices at ICS. The research team agreed to define poorly controlled T2DM patients as those who had HbA1c levels ≥9%. This is because at the time of the protocol writing, local and international guidelines were recommending the initiation of insulin therapy in type 2 diabetes when HbA1c levels were between 8.5 and 10% [32, 33]. Due to this the lack of agreement, the ≥9% cut-off was chosen in accordance with the AACE Comprehensive Diabetes Management Algorithm 2013 [34], which is actually a value in between all recommendations. Moreover, our population characteristics and primary health care patterns of treatment were taken in account. Indeed, those subjects with HbA1c levels > 9% are usually the ones that most often need to be treated with insulin, but this therapy is frequently delayed or not even started by some general practitioners in our primary health-care centres [4]. Some studies report that a multifactorial target intervention used for the management of T2DM is effective and feasible in clinical practice. An intensive intervention strategy delivered at the clinical level is associated with a significant and durable improvement in HbA1c and major cardiovascular risk factors, well beyond that achieved with the usual practices [35]. Some studies describe that patient-centred care is more valuable when targeted to patients with HbA_1c_ > 8.5%. Patient-centred care was most effective and cost-effective in those patients, resulting in more improved glycaemic and metabolic control but also improving quality-adjusted life years and costs of associated complications [36].

In addition to the implementation of methodological tools proposed by the INTEGRA study, identifying the factors that hinder the metabolic control of these patients will allow us to define new strategies for improving patient care, especially for poorly controlled patients. These strategies include improving adherence to treatment proposed by primary care professionals, as well as eliminating the clinical inertia of these professionals treating patients who have difficulty achieving good glycaemic control targets.

There are some limitations in this study. It is well known that patients who voluntarily participate in research projects tend to be more motivated, which can be a common bias in all clinical trials where patients sign an informed consent. In the present study, we aim to recruit a large group of patients in each centre.

The external validity of the study will be determined by the ability to recruit as many candidates as possible because the profiles of the patients included in the study show characteristics of low participation in clinical trials and less adherence to treatment recommendations. An important strength of the study is that it will follow the CONSORT Statement for the reporting and publishing of clinical trials. Another strength of the study is that the Medical Research Council’s methodology allows for the adaptation of the complex intervention’s components to the needs of participants based on the results of the qualitative study, and therefore an easier implementation in Primary care centres.

Targeting interventions at the high-risk population may allow for better use of resources, reduction of cost, and reduction of side effects by avoiding unnecessary use of medications.

## Additional file

Additional file 1: Monitoring plan. The monitoring plan include process for monitoring activities of the study, definition of the key information concerning the realization of the study, the verification of data sources, essential documents of the study. (PDF 294 kb)

## Abbreviations

AQuAS: Agency Qualitat i Avaluació Sanitaries de Catalunya
SIDIAP: Sistema d´Informació per al desenvolupament de la Investigació en Atenció Primària
BMI: Body mass index
DTSQ: Diabetes Treatment Satisfaction Questionnaire
e-CRF: Electronic Case Report Form
ESDQoL: Spanish version of the Diabetes Quality Of Life Questionnaire (ESDQoL)
HbA1c: Glycated haemoglobin
ICH: International Council for Harmonisation of Technical Requirements for Pharmaceuticals for Human Use
IG-1: Intervention Group 1
IG-2: Intervention Group 2
LDL-C: low-density lipoprotein cholesterol
Non-HDL-C: non-high density lipoprotein cholesterol
PIS: Patient Information Sheet
ICF: Informed Consent Form
T2DM: Type 2 Diabetes Mellitus
