# Supplementary material for: INTEGRA study protocol: primary care intervention in type 2 diabetes patients with poor glycaemic control
Source: BMC Fam Pract. 2019 Feb 7;20:25. doi: 10.1186/s12875-019-0916-9 (PMC6367799; doi:10.1186/s12875-019-0916-9)
Supplement: Supplementary file 1 — Monitoring plan. The monitoring plan include process for monitoring activities of the study, definition of the key information concerning the realization of the study, the verification of data sources, essential documents of the study. (PDF 294 kb) [file 12875_2019_916_MOESM1_ESM.pdf]

# MONITORING PLAN

## INTEGRA study: Primary Care Intervention in Type 2 Diabetes Patients with Poor Glycaemic Control

Code: 4R14/047.

ClinicalTrials.gov ID: P14/129

Promoter: Institut Universitari d'Investigació en Atenció Primària Jordi Gol (Barcelona)

Version: 1.0 02 January 2015

# INDEX


1) **SIGNATURE PAGE** .......... 3

2) SPECIFIC MONITORING PLAN FOR THE STUDY .......... 4

2.1) Activities of the monitors .......... 4

2.1.1 Activities prior to the start of the study .......... 5

2.1.2 Initial visits .......... 6

2.1.3 Monitoring visits .......... 6

2.1.3.2 Online monitoring visits .......... 6

2.1.3.3 On-site monitoring visits .......... 7

2.1.4. Management of queries (clarifications and inconsistencies) .......... 8

2.1.5. Management of the serious deviations to the study protocol .......... 8

2.1.6 Closing visit .......... 9

2.2) Reports initial, monitoring and closure visits .......... 9

2.3) frequency of visits to the centres .......... 10

2.4) The trial master file .......... 10

**2.5) Investigator file** .......... 10

3) COMMUNICATION .......... 11

3.1) Routes of communication .......... 11

3.1.1. Site investigators: .......... 11

3.1.2. Research coordinators: .......... 11

3.1.3. Responsible for regulatory affairs: .......... 11

3 .1.4 Responsible data management: .......... 11

3.1.5 Responsible for statistics: .......... 11

3.2 Contact details .......... 12

4.) Audits .......... 13

1) **SIGNATURE PAGE**

Principal investigator

Dr. Angels Mollo

CAP Cervera (Lleida) ___02/01/2015____ ________________

Date signature

Promoter

Dr. Bonaventura Bolíbar Ribas

IDIAP Jordi Gol

Institut de Investigació en

Atenció Primària (IDIAP) Jordi Gol _02/01/2015___ ________________

Date signature

## 2) SPECIFIC MONITORING PLAN FOR THE STUDY

The objectives of this section are the following:

To establish the process for monitoring activities of the study, definition of the key information concerning the realization of the study, the verification of data sources, essential documents of the study and the way of their mutual circulation.

To define the distribution of activities between the monitor and the research coordinator, and the communication circuit between them.

### 2.1) Activities of the monitors

Monitors will conduct monitoring visits to the centers where tasks of supervision of the clinical trial will be performed in accordance with good clinical practice standards and the standard operating procedures (SOPs). In particular they will be responsible for:

- Providing main contact between all researchers involved in the study.
- Verify that the research team follows the directions of the study protocol and complies with standards of good clinical practice and the current legislation.
- Verify that the written informed consent of each subject included in the study has been obtained prior to enrollment into the study.
- Perform and report initial, intermediate and closing visits.
- Collect the information and essential documents of the study sites, including the documentation required for authorizations to the ethical committee.
- Keep and verify the investigator site files, according to the section 8 of the ICH good clinical practice guidelines.
- Reviewing of the electronic case report form (eCRF) online prior to visits and during the study to verify its integrity and consistency, as well as perform data source verification at the center during monitoring visits.
- Make a follow-up of the emission, response and resolution of the requests of clarifications to the eCRF.

- Ensure that all centers have all the necessary materials for the study, and reconcile the surpluses once the study is completed.

- Ensure that the trial master file of the study is complete and remains full.

- Communicate deviations of the Protocol, according to good clinical practices (GCP) and current legislation to the research coordinators and site investigators and taking the appropriate measures to prevent a recurrence of the deviations detected.

### 2.1.1 Activities prior to the start of the study

Objectives:

Establish communication with the investigator team at the centre, in order to identify contact persons and evaluate their feasibility and interest for participation in the study.
Obtain all the documentation necessary requested for approval of the study.

Activities include (but are not limited to):

- Request for documentation required by the Ethics Committee from the study site.
- To determine the competence of the investigator team and adequacy of the site facilities.
- Performing training for good clinical practice
- Review of the Protocol of the study
- Requiring for estimations of recruitment rate in the study site.

Some of the activities may be conducted during the investigators meetings.

### 2.1.2 Initial visits

Objectives:

To ensure that the study files are created before the start of the study, and contains all the required essential documents and it's stored securely on the study site.

Ensure that all the materials needed for the study are available and that the eCRF is accessible and functional. Ensure that authorized personnel know how to access the eCRF and know how to use it.

Ensure that all participating personnel know the Protocol of the study, its procedures, the ethical principles and good clinical practice.

Ensure that the Centre has all the necessary authorizations for the starting with the study before inclusion of the first patient.

Activities include (but are not limited to):

- Session of training for the study staff (Protocol, GCP and eCRF)
- Review of the materials, and the study file
- Report of the activities which shall be sent to the research coordinator

### 2.1.3 Monitoring visits

#### 2.1.3.2 Online monitoring visits

Objectives:

Ensure the correct completion of the eCRF and detect errors or omissions of data.

The activities of the online monitoring include (but are not limited to):

- To review the eCRF prior to the on-site monitoring visit in order to verify the update of the data
- To ensure the correct data entry of the variables of the study

**2.1.3.3 On-site monitoring visits**

Objectives:

To ensure that the study is carried out under the principles of good clinical practice and that study staff is following the directions of the study protocol.

The first on-site visit will be performed for each participating study site right after the inclusion of first 10 patients this visit will be performed to ensure that study stuff fallow study protocol.

The activities include (but are not limited to):

• Review of the documentation of the site study investigators that were present on the initial visit.

• Verification that investigator file is updated correctly and kept safe and appropriate in the study site.

• Review of the conduction of the trial and following of the Protocol by the site investigator team

• Revision of the list of inclusion of patients and the essential documentation for the study, and ensuring that informed consent from the included patients were obtained correctly and before enrolment or any procedure of the study.

• Verification of data

Will be reviewed:

-Criteria for inclusion / exclusion and basal characteristics of the patient.

- Verification of the accuracy and integrity of the data in the eCRF.

- Review of all variables, with the exception of the surveys

- The existence of all written informed consents will be reviewed

- Review of the therapeutic changes or withdrawals of treatment

- Check the record of compliance in the eCRF

### 2.1.6 Closing visit

Objectives:

Ensure that at the end of the trial all the essential study generated documents are archived.

The activities of the visit include (but are not limited to):

- Monitoring of pending patients for verification with data source
- Review of the study site file
- Reconcile the number of patients screened / included / finished
- Verify that all requests for clarification have been resolved
- Inform about the time of archiving of study site file
- Making a report on the closing visit.

### 2.2) Reports initial, monitoring and closure visits

The monitor, after performing on-site visits in the participating centres, will ellaborate appropriate report for initial, monitoring or closing visit.

Register of deviations from the study in every centre will be a document.

The monitor assigned to each centre will make a report for each monitoring visit.

The master study file will contain all those reports of monitoring from different centres participating in the study.

### 2.3) Frequency of visits to the centres

The following plan of visits in each centre is expected to be performed during the study:

1 initial study visit

Monitoring online visits: visits will be carried out at least every 10 days.

On site monitoring visits: these will be performed according to the recruitment rate.

Closing visit: at the end of the study or after the last visit of the last patient of the study at the Centre.

### 2.4) The trial master file

All the essential clinical trial documents will be stored in a trial master file of the study, whose safe and full conservation will be ensured according to the legislation and will be available to the authority if it is requested.

### 2.5) Investigator file

The documentation of the study for each center participating will be organized and filed by site investigators, will be their responsibility for adequate conservation protection according to the existing legislation and good clinical practice guidelines.

## 3) COMMUNICATION

### 3.1) Routes of communication

It is established the following ways of communication:

3.1.1. **Site investigators:**

With the monitors or the research coordinators

**3.1.2. Research coordinators:**
With monitors or the responsible of the IDIAP Jordi Gol

**3.1.3. Responsible for regulatory affairs:**

With the responsible of the IDIAP Jordi Gol

**3 .1.4 Responsible data management:**
With the monitors.

If necessary, with the research coordinators and/or responsible of the IDIAP.

**3.1.5 Responsible for statistics:**
Researchers coordinators or if needed with the responsible of the IDIAP Jordi Gol or monitors.

### 3.2 Contact details

| | | |
|---|---|---|
| **Research coordinators** | | |
| CAP Cervera (Lleida)<br><br>Hospital Germans Trias i Pujol | Angels Mollo<br><br>Didac Mauricio | angelsmollo@gmail.com<br><br>didacmauricio@gmail.com |
| **Biometría**<br><br>*CRD electrónico, centro de gestión de datos* | | |
| IDIAP | Bogdan Vlacho<br><br>Ester Rubinat | bvlacho@idiapjgol.org<br><br>rubinatesther@gmail.com |
| W3 ICF UAB CAT | Joan Vigó | jmv@icf.uab.cat |
| **Statistical analysis**<br><br>**Statistical analysis plan and report** | | |
| Institut Hospital del Mar d'Investigacions Mèdiques | Maria Grau | |
| **Regulatory Affairs**<br><br>**Correspondence with authorities and CEICs** | | |
| IDIAP | Pau Moreno Nogué<br><br>Bogdan Vlacho | pmoreno@idiapjgol.org<br><br>bvlacho@idiapjgol.org |
| **Monitoring**<br><br>**Follow-up of the study and verification of the compliance of good clinical practice** | | |
| IDIAP | Bogdan Vlacho<br><br>Ester Rubinat | bvlacho@idiapjgol.org<br><br>rubinatesther@gmail.com |
